# Importance of Education about Cervical Cancer and Its Preventive Measures for the Promotion of HPV Vaccine According to the WHO Strategies

**DOI:** 10.3390/vaccines9101199

**Published:** 2021-10-18

**Authors:** Yutaka Ueda, Etsuko Miyagi

**Affiliations:** 1 Department of Obstetrics and Gynecology, Graduate School of Medicine, Osaka University, Osaka 565-0871, Japan; 2 Department of Obstetrics and Gynecology, School of Medicine, Yokohama City University, Yokohama 236-0004, Japan; emiyagi@yokohama-cu.ac.jp

The World Health Organization (WHO) has officially declared a global strategy regarding the Elimination of Cervical Cancer in November 2020, which focuses on preventing cervical cancer through HPV vaccination, screening precancerous lesions and managing and treating invasive cervical cancer [1]. The strategies are as follows: “ninety percent of girls should be fully vaccinated by 15 years of age, 70% of women should be screened at least twice with a high-performance test by age 45 and 90% of women with precancer or cancer should receive the appropriate care and treatment, including palliative care”. A simulation model study [2] predicted that the high HPV vaccination coverage of girls can lead to cervical cancer elimination in most lower–middle-income countries by the end of the century. Screening with high uptake will expedite reductions and will be necessary to eliminate cervical cancer in countries with the highest burden.

According to a previous report [3], 107 (55%) of the 194 WHO Member States introduced HPV vaccination as of June 2020. HPV vaccine has been approved in 88% of high–income countries for females and in 44% for males. Even in low-to-middle-income countries, the vaccine has been approved in 40% and 5% for females and males, respectively.

In this special edition of “Latest National HPV Vaccine Programs and Outcomes in the World”, the status of the global HPV vaccine program and its coverage is demonstrated from various countries. The editors believe that knowing the status of each country may accelerate and support the efforts to meet the goals imposed by WHO and expect that this Special Issue will play a certain role in reaching the goal.

First, Dr. Diana Wangeshi Njuguna et al. [4] show the knowledge, attitudes and practices of main stakeholders regarding HPV infection and HPV vaccination in Kenya. HPV vaccination was introduced in Kenya in 2019. According to their semi-structured interviews from girls and boys, their parents, teachers and community leaders, it was shown that a significant proportion of girls and boys have little knowledge of HPV infection and HPV vaccine. Most parents demonstrated negative attitudes toward HPV vaccination for boys. Involving the community by raising awareness, changing negative beliefs about the HPV vaccine and promoting HPV vaccine perceptibility was thought to promote HPV vaccination.

Another group show the situation in Japan. In Japan, subsidies from local and national governments for the HPV vaccination program for girls aged 13–16 years commenced in 2010. In 2013, HPV vaccination became a national immunization program in girls aged 12–16 years. However, in June 2013, the Japanese government decided to suspend the governmental recommendation for the HPV vaccine. The rate of HPV vaccination decreased drastically, from 70% to almost zero. This situation has substantially influenced the lower awareness about cervical cancer prevention, even among medical school students [5]. However, even in such a situation, a brief web-based educational intervention was shown to increase the willingness of adults to consider the HPV vaccine for their children, especially among men in Japan [6]. Providing adequate information is thought to be a useful strategy to improve the currently low rates of HPV vaccination. We believe that an international alliance will change the HPV vaccine status in Japan.

Other authors present various situations in their respective countries. We need to understand each other’s situations in countries where the HPV vaccine has been successfully disseminated and in countries where it has not been well disseminated, and in this Special Issue, we realize the various steps necessary for increasing of uptake of HPV vaccination. The dissemination of correct knowledge is essential in any country and in any situation. We hope that this special edition of “Latest National HPV Vaccine Programs and Outcomes in the World” will contribute to the improvement of the health of people worldwide.

## Data Availability

Data sharing not applicable to this article as no datasets were generated or analysed during the current study.
